# How should I treat patients with subclinical atrial fibrillation and atrial high-rate episodes? Current evidence and clinical importance

**DOI:** 10.1007/s00392-022-02000-7

**Published:** 2022-03-15

**Authors:** Fabienne Kreimer, Andreas Mügge, Michael Gotzmann

**Affiliations:** University Hospital St Josef-Hospital Bochum, Cardiology and Rhythmology, Ruhr University Bochum, Gudrunstraße 56, 44791 Bochum, Germany

**Keywords:** Subclinical atrial fibrillation, Atrial high-rate episodes, Cardiac implantable electronic devices, Ischemic stroke

## Abstract

Long-term and continuous ECG monitoring using cardiac implantable electronic devices and insertable cardiac monitors has improved the capability of detecting subclinical atrial fibrillation (AF) and atrial high-rate episodes. Previous studies demonstrated a high prevalence (more than 20%) in patients with cardiac implantable electronic devices or insertable cardiac monitors. Subclinical AF and atrial high-rate episodes are often suspected as the cause of prior or potential future ischemic stroke. However, the clinical significance is still uncertain, and the evidence is limited. This review aims to present and discuss the current evidence on the clinical impact of subclinical AF and atrial high-rate episodes. It focuses particularly on the association between the duration of the episodes and major clinical outcomes like thromboembolic events. As subclinical AF and atrial high-rate episodes are presumed to be associated with ischemic strokes, detection will be particularly important in patients with cryptogenic stroke and in high-risk patients for thromboembolism. In this context, it is also interesting whether there is a temporal relationship between the detection of subclinical AF and atrial high-rate episodes and the occurrence of thromboembolic events. In addition, the review will examine the question whether there is a need for a therapy with oral anticoagulation.

**Graphical abstract:**

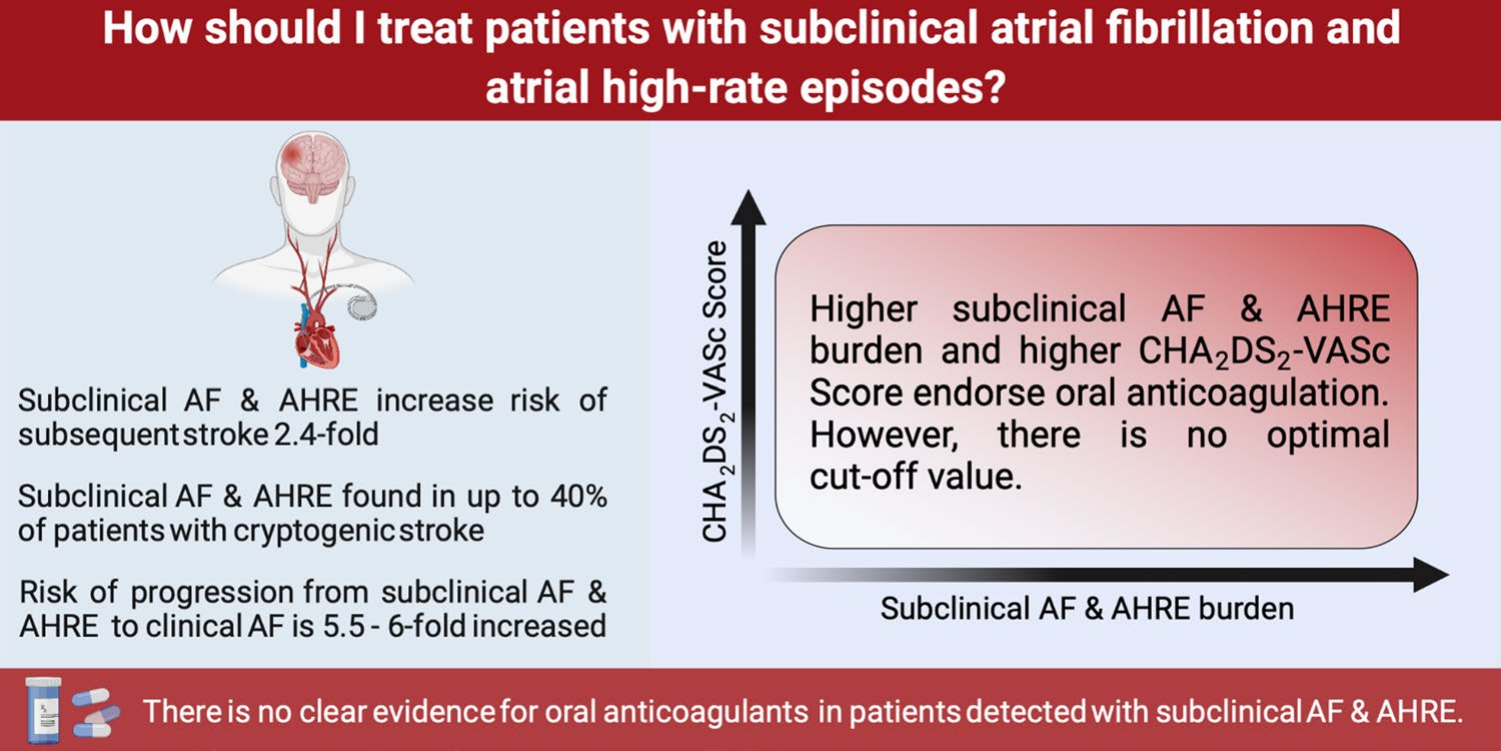

## Introduction

Atrial fibrillation (AF) is a supraventricular tachyarrhythmia accompanied by uncoordinated atrial electrical excitation and consequent ineffective atrial contraction [1]. With a worldwide estimated prevalence of 2–4%, AF is the most common cardiac arrhythmia in adults and is associated with increased overall morbidity and mortality. The lifetime risk of AF is currently one in three for a 55-year-old individual of European ancestry. Due to the lengthening survival of the general population and intensified diagnostic testing for suspected AF, the prevalence is expected to increase significantly [1–5]. The etiology of AF has not yet been conclusively determined, but a complex interplay of genetic and environmental risk factors is hypothesized, predominantly in the presence of cardiac (e.g., valvular heart disease, coronary artery disease, congestive heart failure) and extracardiac diseases (e.g., hyperthyroidism, electrolyte disturbances, drug-toxic influences) [1]. Electrocardiogram (ECG) documentation is mandatory for the diagnosis of AF. By convention, an ECG episode of at least 30 s without detectable P waves and with irregular RR intervals is diagnostic for AF [1]. AF is classified according to frequency and duration of occurrence into initially diagnosed, paroxysmal, persistent, long persistent, and permanent AF [1].

Patients with AF may suffer from many possible symptoms such as palpitations, dyspnea, or fatigue. However, several studies revealed that a considerable proportion of patients present asymptomatic. Asymptomatic AF is more difficult to diagnose and delayed diagnosis can cause complications such as thromboembolism [6–11].

In 20–40% of ischemic strokes, the cause remains initially unexplained [12]. There is a cryptogenic stroke when no definite cause could be detected in patients with an ischemic stroke despite extensive diagnostic workup. In approximately 20–30% of patients with ischemic stroke and 10% of patients with cryptogenic stroke, AF is present or diagnosed during the course [1]. Cardioembolic strokes associated with AF are usually more severe and often recur [13–15]. Therefore, an early diagnosis of AF and initiation of therapy with oral anticoagulants are elementary. However, it is often complicated by the lack of symptoms and noncontinuous occurrence of AF. Subclinical AF is often suspected as the cause of cryptogenic stroke.

In recent years, several studies have been undertaken to improve the detection of AF. At the same time, patients with cardiac implantable electronic devices or insertable cardiac monitors have become the focus of interest, as continuous rhythm monitoring is possible in these patients [12, 16–20]. The prevalence of atrial high-rate episodes and subclinical AF in these patients is high (20–70%) but the clinical implications are uncertain [11, 21–23].

**Fig. 1:**
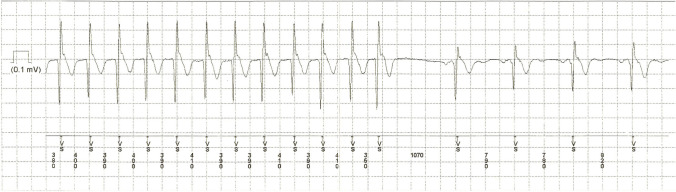
Device query showing an atrial high-rate episode

**Fig. 2:**
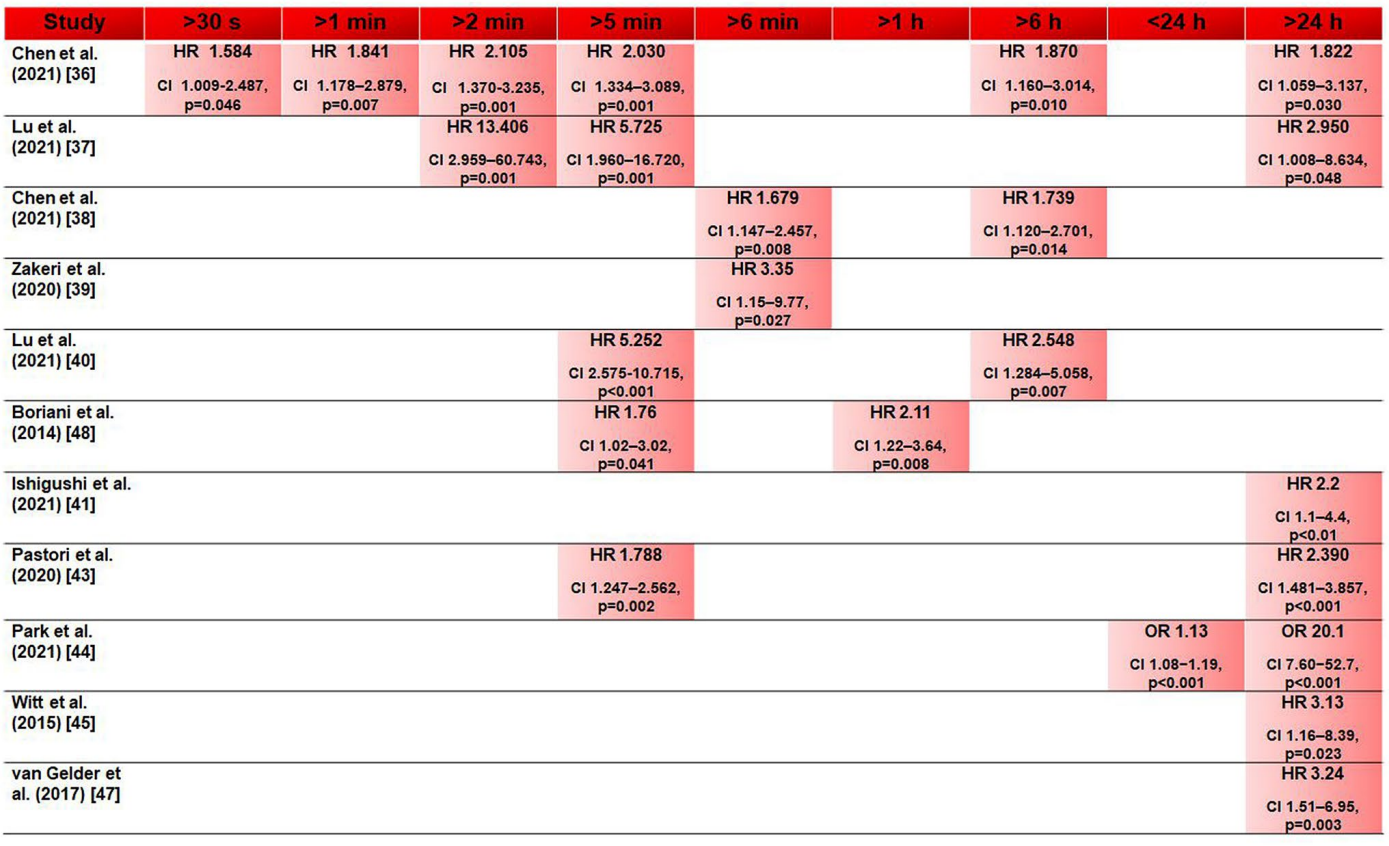
Optimal threshold values for the duration of subclinical atrial fibrillation and atrial high-rate episodes for an increased thromboembolic risk in selected studies (with indication of the hazard ratio (HR)/odds ratio (OR))

The terms subclinical AF and atrial high-rate episodes are sometimes defined differently and used as a synonymous which may affect the comparability of previous studies [22]. Both phenomena have in common that they are asymptomatic episodes identified and confirmed by ECG obtained from cardiac devices and monitors in patients without the history of AF. In a “Scientific Statement from the American Heart Association”, atrial high-rate episodes are defined as device-detected atrial events, usually tachyarrhythmias, meeting programmed or other specified atrial high-rate criteria (usually ranging between 175 and 220 bpm) [24]. Subclinical AF is defined as asymptomatic episodes of AF detected and confirmed by cardiac devices and monitors and not previously detected by electrocardiographic or ambulatory monitoring [24]. Subclinical AF comprises atrial high-rate episodes affirmed to be AF, atrial flutter, or atrial tachycardia. The current ESC guidelines state that both phenomena must be confirmed by visually verified intracardiac electrograms or ECG-recorded rhythms, as some episodes may be electrical artifacts/false-positive signals [1] (Figs. 1, 2).

In contrast to this, clinical AF is defined as symptomatic or asymptomatic AF documented by 12-lead surface ECG [1].

Boriani et al. [21] pointed out that the thromboembolic risk increased with increasing duration of atrial high-rate episodes. However, they raised awareness for the heterogeneity of physicians’ clinical management when atrial high-rate episodes are detected. The meta-analysis of Mahajan et al. [25] demonstrated that subclinical AF led to a 2.4-fold increase in stroke risk (HR 2.41, 95% CI 1.78–3.26). Furthermore, the risk of subsequent clinical AF increased significantly when subclinical AF was detected (HR 5.66, 95% CI 4.02–7.97). Recently, these findings were confirmed by Vitolo et al. [26] whose meta-analysis had the additional advantage of focusing only on patients without a history of clinical AF. They noticed a risk ratio for thromboembolic events in patients with atrial high-rate episodes of 2.13 (95% CI 1.53–2.95) and a risk ratio for subsequent clinical AF of 3.34 (95% CI 1.89–5.90). Nevertheless, it is currently ambiguous whether each episode of subclinical AF/atrial high-rate episodes is associated with an increased risk of stroke and whether initiation of a therapy with oral anticoagulants is indicated.

**Fig. 3:**
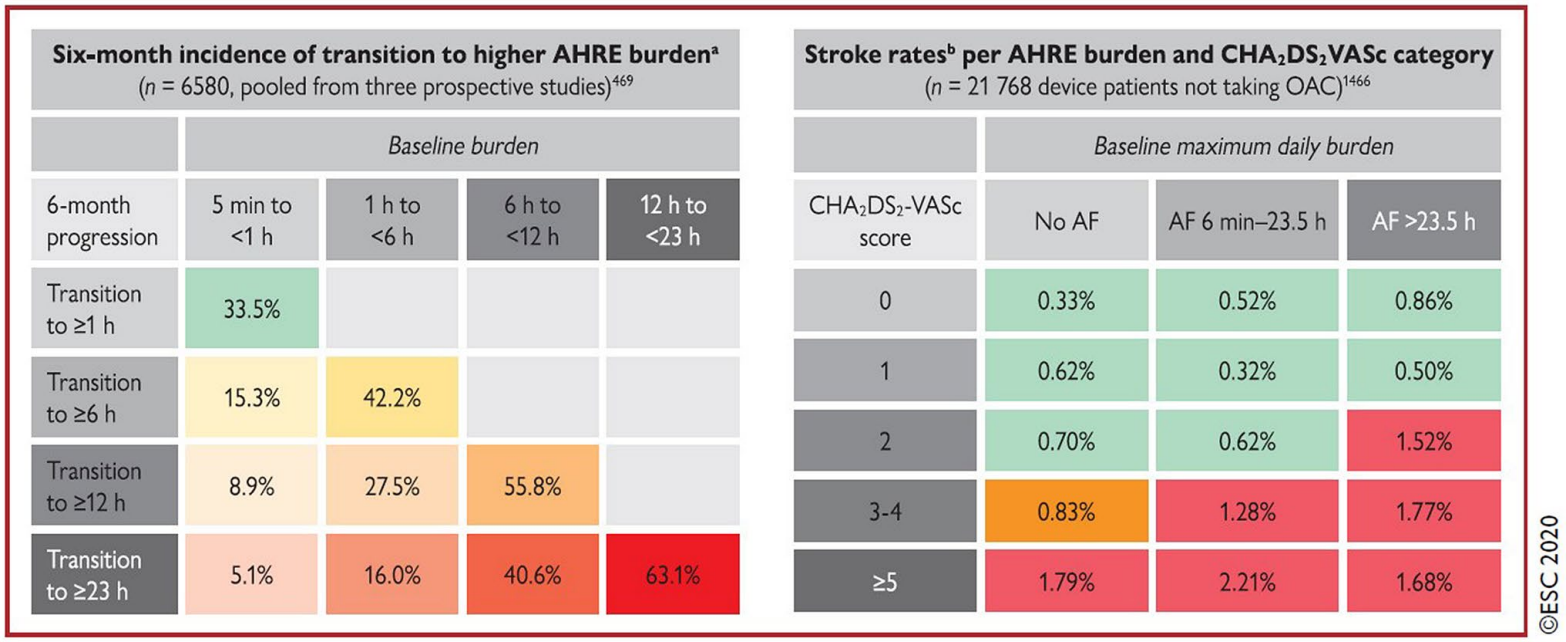
**a** 6-month progression of AHRE burden depending on AHRE baseline burden. The higher the burden at diagnosis, the greater the likelihood of transition to a higher burden in the following 6 months. **b** Risk of stroke depending on AHRE baseline burden and CHA2DS2-VASc score. Stroke rates with indication for OAC are shown in red. *AHRE*  atrial high-rate episodes, *CHA2DS2-VASc *congestive heart failure, hypertension, age ≥ 75 years, diabetes mellitus, stroke, vascular disease, age 65–74 years, sex category (female), *OAC* oral anticoagulant. From reference 1, with permission

An individualized decision-making using a management algorithm is required in all patients with atrial high-rate episodes to evaluate their risk of stroke [21–23]. The CHA2DS2-VASc score (congestive heart failure, hypertension, age, diabetes mellitus, prior stroke or TIA or thromboembolism, vascular disease, age, and sex category) has been used for many years to assess the risk of stroke in patients with clinical AF [1]. However, the significance of the CHA2DS2-VASc score in patients with a low burden of subclinical AF or atrial high-rate episodes is ambiguous. In awareness of a possible progression to a higher burden or clinical AF, the ESC guidelines suggest risk stratification based on the duration of arrhythmia and the CHA2DS2-VASc score (Fig. 3) [1]. The current ESC guidelines recommend in patients with subclinical AF/atrial high-rate episodes a complete cardiovascular evaluation with ECG recording, clinical risk factors/comorbidity evaluation, and thromboembolic risk assessment using the CHA2DS2-VASc score. Moreover, continued patient follow-up and monitoring are recommended to detect a progression to clinical AF, monitor the subclinical AF/atrial high-rate episodes burden and detect changes in underlying clinical conditions [1].

This review aims to outline the current evidence with a focus on the impact of duration of subclinical AF/atrial high-rate episodes. In addition, the temporal relationship between subclinical AF/atrial high-rate episodes and thromboembolic events and the meaning in cryptogenic stroke and high-risk patients will be evaluated. Furthermore, the risk of progression to clinical AF will be elaborated. Within the scope of this, the value of oral anticoagulant therapy will also be discussed.

### Association between subclinical atrial fibrillation and atrial high-rate episodes, and the risk of stroke

The landmark ASSERT trial [27] examined 2580 nonanticoagulated patients with cardiac implantable electronic devices. During a 3-month monitoring period after device implantation, subclinical AF was detected in 10.1% of patients and any episode of subclinical AF lasting > 6 min was associated with a 2.5-fold increase in the risk of subsequent stroke.

The TRENDS study [28] demonstrated that subclinical AF > 5.5 h per day on any of 30 prior days doubled the risk of stroke compared to patients with no subclinical AF or a lower subclinical AF burden. Glotzer et al. [29] revealed in an analysis of the MOST trial that atrial high-rate episodes detected by a pacemaker were associated with more than twice the risk of death and stroke. More recent studies have also demonstrated a significant association between the detection of subclinical AF/atrial high-rate episodes and the occurrence of thromboembolic events [30–32].

On the contrary, Li et al. [33] demonstrated in a study including 594 patients with cardiac implantable electronic devices that atrial high-rate episodes were not independently associated with thromboembolic events. The authors pointed out that comorbidity burden had a greater impact on the occurrence of thromboembolic events than atrial high-rate episodes by themselves.

It should be noted that the differences in subclinical AF/atrial high-rate episodes burden and in composition of the study populations may affect the comparability of these studies.

However, in consideration that some studies have demonstrated an association between subclinical AF/atrial high-rate episodes and stroke, initiation of oral anticoagulant therapy in patients with subclinical AF/atrial high-rate episodes is discussed controversially. The evidence on the significance of subclinical AF/atrial high-rate episodes and on possible benefits of a therapy with oral anticoagulants in these patients is still doubtful.

### Duration of subclinical atrial fibrillation and atrial high-rate episodes, and the risk of thromboembolic events

An important question is whether longer episodes of subclinical AF/atrial high-rate episodes are associated with a higher incidence of thrombotic events. Very brief episodes of subclinical AF/atrial high-rate episodes (< 15–20 s) are considered clinically irrelevant because they are not significantly associated with longer episodes or increased risk of thromboembolic events [34]. Nevertheless, the meta-analysis of Sagris et al. [35] indicated recently that even short atrial high-rate episodes lasting ≥ 30 s increased the risk of thromboembolism (HR 4.41, 95% CI 2.32–8.39), which remained constant at higher durations (5 min, 6 and 24 h). However, the risk of thromboembolism was higher in patients with cumulative atrial high-rate episodes lasting ≥ 24 h compared to those with shorter duration or no atrial high-rate episodes (HR 1.25, 95% CI 1.04–1.52).

To date, there are few data on an optimal cutoff value at which subclinical AF/atrial high-rate episodes are clinically relevant and thus in need of treatment. Considering the possible progression to a higher arrhythmia burden, it is even difficult to define an optimal cutoff value.

Only a few studies exist that evaluated the association of subclinical AF/atrial high-rate episodes duration and subsequent thromboembolic events. These studies mostly compared the clinical outcome of patients with cardiac implantable electronic devices with very low subclinical AF/atrial high-rate episodes burden (< 6 min), low burden (> 6 min to 6 h), intermediate burden (> 6 h to 24 h) and higher burden (> 24 h) [36–47] (Table 1).

**Table 1:** Duration of subclinical atrial fibrillation and atrial high-rate episodes, and the risk of thromboembolic events

Study/first author (year)	Study design	*n*	SCAF/AHRE duration	Recommended cutoff value
Chen et al (2021) [36]	Retrospective cohort study	355	30 s, 1 min, 2 min, 5 min, 6 h, 24 h	1 min
Lu et al (2021) [37]	Retrospective cohort study	355	2 min, 5 min, 6 h, 24 h	2 min
Chen et al (2021) [38]	Retrospective cohort study	470	6 min, 6, h, 24 h	6 min
Zakeri et al (2020) [39]	Analysis of the multicentre, prospective, randomized REM-HF trial	1561	6 min, 24 h	6 min
Lu et al (2021) [40]	Retrospective cohort study	481	5 min, 6 h, 24 h	5 min
Boriani et al (2014) [48]	Pooled analysis of 3 prospective observational studies	10,016	5 min, 1 h, 6 h, 12 h, 23 h	1 h
Ishiguchi et al (2021) [41]	Retrospective cohort study	710	< 6 min, 6 min, 24 h	24 h
Pastori et al (2020) [43]	Retrospective cohort study	852	5 min, 24 h	24 h
Park et al (2021) [44]	Prospective observational study	496	< 24 h, 24 h	24 h
Witt et al (2015) [45]	Retrospective cohort study	394	6 min, 24 h	24 h
Li et al (2021) [46]	Retrospective cohort study	500	24 h	24 h
van Gelder (2017) [47]	Analysis of the multicentre, prospective, randomized ASSERT trial	2580	6 min, 6 h, 24 h	24 h

*SCAF* subclinical atrial fibrillation, *AHRE* atrial high-rate episodes

Recently, Chen et al. [36] demonstrated that all durations of atrial high-rate episodes detected by pacemaker (> 30 s to 24 h) were associated with subsequent cardiovascular and cerebrovascular events. Nevertheless, they identified an optimal atrial high-rate episodes cutoff value of 1 min. Another study also demonstrated that even a very short duration of atrial high-rate episodes has impact on the occurrence of neurological events: Lu et al. [37] revealed an optimal atrial high-rate episodes cutoff value of 2 min. They examined a study cohort of 355 patients with pacemaker over a mean follow-up time of 42 months and showed that atrial high-rate episodes lasting > 2 min, but also > 5 min were significantly associated with neurological events.

There are two studies that demonstrated a significant association between the occurrence of thromboembolic events and atrial high-rate episodes at a duration of at least 6 min and less than 24 h. Chen et al. [38] investigated 470 patients with cardiac implantable electronic devices with regard to subsequent thromboembolic events after atrial high-rate episodes detection. The optimal atrial high-rate episodes cutoff value was 6 min. Whereas atrial high-rate episodes lasting > 6 min to 24 h were independently associated with thromboembolic events, this was not the case for durations > 24 h when the CHA2DS2-VASc score was excluded as a confounder. The same applied for the study of Zakeri et al. [39] who noticed the highest stroke risk for patients with subclinical AF lasting > 6 min to 24 h compared to patients with no subclinical AF or subclinical AF lasting > 24 h. Among patients without AF in history, subclinical AF lasting > 6 min implied a three-times higher stroke risk. In line with these findings, Lu et al. [40] proved in 481 patients with pacemaker that atrial high-rate episodes lasting > 5 min and > 6 h were independently associated with thromboembolic events, but not atrial high-rate episodes lasting > 24 h. Boriani et al. [48] conducted a pooled analysis of three prospective studies including 10,016 patients. An AF burden of at least 1 h was associated with the highest risk for ischemic stroke while AF burden of ≥ 6, ≥ 12, and ≥ 23 h did not reach statistical significance Fig. 1.

The results of the latter studies seem paradoxical, as it was assumed that a higher AF burden was associated with a worse outcome. Limiting factors of these studies that identified low to intermediate atrial high-rate episodes burden as the optimal cutoff value may have been a relatively small number of patients with thromboembolic events and a lower overall number of patients with atrial high-rate episodes lasting > 24 h. Nevertheless, these studies may indicate that there is not necessarily a linear relationship between the duration of AF and the risk of a thromboembolic event.

By contrast, the most studies that evaluated the subclinical AF/atrial high-rate episodes burden regarding to the occurrence of subsequent thromboembolic events proved the highest risk when subclinical AF/atrial high-rate episodes lasted at least for 24 h. Recently, Ishiguchi et al. [41] showed in a retrospective analysis of 710 patients with cardiac implantable electronic devices that a rising atrial high-rate episodes burden was associated with an increased risk of stroke. They were able to demonstrate that the risk of stroke was higher in the group with atrial high-rate episodes lasting > 24 h than in the group with atrial high-rate episodes lasting < 6 min. However, they also indicated that a higher atrial high-rate episodes burden led to a higher rate of major bleedings due to an increased likelihood of oral anticoagulant prescription in the presence of longer lasting atrial high-rate episodes.

The study by Pastori et al. [43] confirmed the described findings by identifying a significant higher incidence rate of major adverse cardiovascular events in patients with atrial high-rate episodes lasting > 24 h. Although there was also an association between atrial high-rate episodes lasting > 5 min and major adverse cardiovascular events, the association was stronger when atrial high-rate episodes lasted at least 24 h. The studies of Park et al. [44] and Witt et al. [45] examined the association of subclinical AF/atrial high-rate episodes and stroke in patients without prior AF. Both studies concluded that atrial high-rate episodes lasting > 24 h had the strongest association with stroke. However, Park et al. only distinguished between no subclinical AF, subclinical AF lasting < 24 h and > 24 h, whereas Witt et al. had an additional group with atrial high-rate episodes lasting > 6 min to 24 h.

An analysis of Li et al. [46] focused on the detection of atrial high-rate episodes lasting > 24 h. 500 nonanticoagulated patients with cardiac implantable electronic devices and without prior AF were analyzed regarding the occurrence of stroke after atrial high-rate episodes lasting > 24 h. The authors were able to demonstrate an association between atrial high-rate episodes lasting > 24 h and an increased risk of all-cause mortality and stroke.

In a secondary analysis of patients in the ASSERT trial, van Gelder et al. [47] evaluated the impact of subclinical AF duration on the appearance of subsequent stroke. They also found that subclinical AF lasting > 24 h was significantly associated with stroke, while the risk of stroke was not different in patients without subclinical AF or subclinical AF lasting > 6 min to 24 h.

Taken all these studies together, the findings indicated that the presence of subclinical AF/atrial high-rate episodes, and particularly a higher burden of subclinical AF/atrial high-rate episodes, is significantly associated with an increased risk of thromboembolic events (Fig. 2; Table 1). Nevertheless, a direct causality cannot be proven. More data are still required to better assess the importance of subclinical AF/atrial high-rate episodes that are highly detected in patients with cardiac implantable electronic devices and insertable cardiac monitors. More detailed analyses dealing with the impact of duration of subclinical AF/atrial high-rate episodes could help to identify high-risk patients who would benefit from a therapy with oral anticoagulants.

### Temporal relationship between subclinical atrial fibrillation and atrial high-rate episodes, and thromboembolic events

Another question of interest is whether there is a temporal relationship between the detection of subclinical AF/atrial high-rate episodes and the occurrence of thromboembolic events (Table 2). The findings could help in decision-making on whether oral anticoagulation should be initiated immediately when atrial high-rate episodes are detected. Moreover, it may be useful to monitor patients for increasing burden of subclinical AF/atrial high-rate episodes or progression to clinical AF.

**Table 2:** Temporal relationship between subclinical atrial fibrillation and atrial high-rate episodes, and thromboembolic events

Study/first author (year)	Study design	n	Thromboembolic events	Temporal relationship
Brambatti et al. (2014) [52]	Analysis of the multicentre, prospective, randomized ASSERT trial	2580	18	4 patients with subclinical AF detected within 30 days before thromboembolic event
Li et al (2019) [33]	Prospective observational study	594	33	8 patients with atrial high-rate episodes prior to thromboembolic events; mean time from first detection to thromboembolism was 20.8 months
Witt et al (2015) [45]	Retrospective cohort study	394	27	10 patients with atrial high-rate episodes detected within 2 months before thromboembolic event

*AF* atrial fibrillation

This question is also of clinical interest as it is assumed that the development of a thrombus in the left atrial appendage requires at least 48 h. This assumption leads to the recommendation that patients do not require thrombus exclusion by transoesophageal echocardiography in the first 48 h after the first occurrence of clinical AF before cardioversion even without pre-existing anticoagulation prophylaxis [1]. While this recommendation has been mentioned in international guidelines for decades, it is based on theoretical rather than evidence-based considerations [49, 50]. However, in 1997, Weigner et al. [50] demonstrated a low risk of thromboembolism (0.8%) following cardioversion in patients with AF that lasted less than 48 h. By contrast, Stoddard et al. [51] reported that 14% of patients with AF ≤ 48 h had left atrial thrombi on transoesophageal echocardiography. The assumption that the development of a thrombus in the left atrial appendage takes at least 48 h, therefore, seems to be associated with considerable uncertainty.

In a secondary analysis of 2580 patients in the ASSERT trial, Brambatti et al. [52] investigated the temporal relationship between the detection of subclinical AF lasting > 6 min and the appearance of stroke. 26 of 51 patients who suffered from thromboembolic events in the follow-up period had detected subclinical AF. 18 patients had subclinical AF before the event and the remaining 8 patients developed subclinical AF after the event. It is worth mentioning that only four patients had subclinical AF within 30 days before the thromboembolic event. In a more detailed analysis of these four patients, it was found that the mean duration of subclinical AF in these patients was > 6 h per day [53].

Li et al. [33] found an atrial high-rate episodes incidence rate of 29.5% in 594 nonanticoagulated patients with cardiac implantable electronic devices. 5.5% of the patients with atrial high-rate episodes suffered from stroke. There was no temporal relationship between the detection of atrial high-rate episodes and the occurrence of stroke. Eight patients had detected atrial high-rate episodes prior to thromboembolic events. For these patients, the mean time from the first detection of atrial high-rate episodes to thromboembolism was 20.8 months.

The study of Witt et al. [45] revealed that 20% of the study population had early detected atrial high-rate episodes within 6 months after cardiac resynchronization therapy implantation. 27 of 394 patients had a thromboembolic event and 10 of these patients had atrial high-rate episodes detected within 2 months before the thromboembolic event.

Overall, there are few data on the temporal relationship between the detection of subclinical AF/atrial high-rate episodes and the occurrence of thromboembolic events. Nevertheless, it should be emphasized that the available studies did not demonstrate a clear temporal association between subclinical AF/atrial high-rate episodes and thromboembolic events (Table 2). It is also noteworthy that most patients did not have atrial high-rate episodes shortly before the thromboembolic event. The available studies suggest that subclinical AF/atrial high-rate episodes may be a manifestation of atrial cardiomyopathy with increased risk of stroke rather than a true cause of thromboembolic events. Furthermore, a temporal relationship between subclinical AF/atrial high-rate episodes and the development of atrial appendage thrombus has never been demonstrated.

### Prevalence of subclinical atrial fibrillation and atrial high-rate episodes in cryptogenic stroke and high-risk patients

Subclinical AF is often suspected to be the cause of cryptogenic stroke. For this reason, diagnostic efforts have been intensified in recent years. Insertable cardiac monitors are often used which have the advantage of long and continuous ECG monitoring. But cardiac implantable electronic devices can detect subclinical AF/atrial high-rate episodes as well. In the past, several studies demonstrated the benefit of insertable cardiac monitors in patients with cryptogenic strokes by means of high subclinical AF detection rates (Table 3).

**Table 3:** Prevalence of subclinical atrial fibrillation and atrial high-rate episodes in cryptogenic stroke and high-risk patients

Study/first author (year)	Study design	n	AF definition	Monitoring duration	Time to AF detection	AF detection rate
CRYSTAL AF (2014) [12]	Multicentre, prospective, randomized study	221	30 s	3 years	84 days	8.9% at 6 months, 12.4% at 12 months
Ziegler et al. (2017) [54]	Prospective observational study	1247	2 min	579 days	112 days	21.5% at 24 months
Cuadrado-Godia et al. (2020) [16]	Prospective and historical cohort study	90	1 min	30 months	51 days	58.5% at 30 months
Ungar et al. (2021) [20]	Multicentre, prospective study	334	5 min	24 months	60 days	22.0% at 6 months, 24.1% at 12 months, 31.5% at 24 months
Kreimer et al. (2021) [18]	Retrospective cohort study	366	30 s	627 days	277 days	20.5% at 627 days
ASSERT-II (2017) [17]	Multicentre, prospective study	256	5 min	16 months	5 months	34.4% per year
REVEAL AF (2017) [19]	Multicentre, prospective study	385	6 min	23 months	123 days	20.4% at 6 months, 27.1% at 12 months, 33.6% at 24 months, 40.0% at 30 months
PREDATE AF (2017) [55]	Single-center, prospective study	245	6 min	451 days	141 days	22.4% at 451 days

*AF* atrial fibrillation

The CRYSTAL AF trial [12] was the largest and first randomized controlled trial on subclinical AF in patients with cryptogenic stroke. 441 patients with previous cryptogenic stroke or transient ischemic attack were randomized 1:1 to either the insertable cardiac monitor group or the control group with conventional follow-up to detect AF lasting ≥ 30 s. Using insertable cardiac monitors, AF was detected in 8.9% of patients by 6 months and in 12.4% of patients by 12 months, compared to 1.4% and 2.0% in the control group, respectively. The median time to AF detection was 84 days.

A few years later, Ziegler et al. [54] conducted a prospective insertable cardiac monitor study including 1247 patients with cryptogenic stroke for the detection of AF. By 2 years, the detection rate of AF was 21.5%, and the median time to first detection was 112 days. These authors also concluded that ECG monitoring by insertable cardiac monitor is superior to conventional follow-up.

Recently, Cuadrado-Godia et al. [16] examined 191 patients with cryptogenic stroke for the detection of subclinical AF lasting > 1 min using insertable cardiac monitors or conventional follow-up. After a mean follow-up of 30 months, the AF detection rates were 58.5% and 21.3% for patients in the insertable cardiac monitor group and the conventional group.

Lower, but still high detection rates were presented in a study of Ungar et al. [20]. The investigators implanted insertable cardiac monitors in 334 patients with cryptogenic stroke. After a mean follow-up of 23.6 months, subclinical AF lasting > 5 min was detected in 27.5% of patients. The subclinical AF burden was 22.0%, 24.1% and 31.5% at 6, 12 and 24 months after insertable cardiac monitor implantation. Subclinical AF was detected after a median time of 60 days.

Recently, we analyzed a study cohort of 366 patients with insertable cardiac monitors with and without previous cryptogenic stroke for the detection of subclinical AF [18]. After a mean follow-up of 627 days, 20.5% of patients were diagnosed with subclinical AF lasting ≥ 30 s. A subgroup analysis revealed that approximately one in four patients with a history of cryptogenic stroke suffered from subclinical AF. The mean time to first detection of subclinical AF was 277 days.

Three prospective studies dealt with the benefit of insertable cardiac monitors for detecting AF in patients at higher risk. The ASSERT-II trial [17] implanted insertable cardiac monitors in 256 patients > 65 years with no history of AF and at least one cardiovascular risk factor. Subclinical AF lasting > 5 min was detected in 34.4% per year in the overall group and in 39.4% per year in patients with a history of thromboembolic event. It is worth mentioning that previous stroke was not a predictor of the detection of subclinical AF. Nevertheless, the authors inferred that a detection of subclinical AF is frequent in older patients without a history of AF.

The REVEAL AF trial [19] was conducted to quantify the incidence of AF in patients at high risk. Insertable cardiac monitors were implanted in 385 patients with a CHA2DS2-VASc score of 2 or greater. The subclinical AF (> 6 min) detection rates were 6.2%, 20.4%, 27.1%, 33.6% and 40.0% at 30 days and 6,12,24 and 30 months, respectively. The median time to first detection of subclinical AF was 123 days. Hence, the investigators concluded that subclinical AF would have gone undetected in most patients with conventional follow-up. The PREDATE AF trial [55] enrolled 245 patients with no history of AF and a CHA2DS2-VASc score ≥ 2 to detect subclinical AF (> 6 min) using insertable cardiac monitors. During a mean follow-up of 451 days, subclinical AF was detected in 22.4% of patients with a mean time to detection of 141 days. Among patients with subclinical AF, 76.4% were prescribed oral anticoagulation.

The studies demonstrated the great frequency of subclinical AF in patients with cryptogenic stroke and patients with high risk for subsequent stroke (Table 3). The findings also suggest that the prevalence of AF is likely much higher than previously thought. However, the studies examined only high-risk patients for AF. Therefore, the results cannot be directly transferred to the overall population. Another important aspect resulting from the studies is the conclusion that the clinical significance of the detection of subclinical AF is still unclear. A higher prevalence of subclinical AF does not necessarily correlate with higher stroke rates. Thus, oral anticoagulation therapy is not always needed in patients with detected subclinical AF.

However, the recently published LOOP study [56] did not show a reduction in thromboembolic events in patients with insertable cardiac monitors compared to patients screened conventionally for AF. Therefore, the therapeutic implications of the above studies are questionable.

### Oral anticoagulation in subclinical atrial fibrillation and atrial high-rate episodes

Evaluating the impact of therapy with oral anticoagulants, we searched for studies that investigated the prescription of oral anticoagulants in patients detected with subclinical AF/atrial high-rate episodes (Table 4).

**Table 4:** Oral anticoagulation in subclinical atrial fibrillation and atrial high-rate episodes

Study/first author (year)	Study design	*n*	Results
Cuadrado-Godia et al. (2020) [16]	Prospective and historical cohort study	90	Prescription of oral anticoagulation in 65.5% of patients with insertable cardiac monitor; stroke recurrence in 3.3% of patients with insertable cardiac monitor
Perino et al (2019) [57]	Retrospective cohort study	10,012	Great practice variation in prescription of oral anticoagulation; prescription after detected atrial high-rate episodes lasting > 24 h was associated with a reduced stroke risk
Sandgren et al (2018) [58]	Retrospective cohort study	678	Prescription of oral anticoagulation in 62% of patients with detected subclinical AF; risk of stroke was 1.9% in patients with detected subclinical AF
Marinheiro et al (2019) [59]	Prospective observational study	923	Thromboembolic events and major bleedings in 4.9% per 100 person-year in patients with oral anticoagulation
LOOP (2021) [56]	Multicentre, prospective, randomized study	1501	Prescription of oral anticoagulation in 29.7% of patients with insertable cardiac monitor; thromboembolic events in 4.5% of patients with insertable cardiac monitor

*AF* atrial fibrillation

The insertable cardiac monitor study of Cuadrado-Godia et al. [16] demonstrated, in addition to a higher detection rate of subclinical AF, that a therapy with oral anticoagulants was prescribed in 65.5% versus 37.6% in the insertable cardiac monitor group versus control group. Moreover, stroke recurrence was noted in 3.3% versus 10.9% in the insertable cardiac monitor group versus control group. The findings illustrated that monitoring with insertable cardiac monitors led to a higher rate of oral anticoagulants prescription and a decrease in stroke recurrence.

Perino et al. [57] showed in a large retrospective study comprising 10,012 patients with cardiac implantable electronic devices that atrial high-rate episodes lasting > 6 min, > 1 h, > 6 h and > 24 h were detected in 45%, 39%, 32% and 24% of patients, respectively. There was a great practice variation in 90-day oral anticoagulant therapy initiation (> 6 min: 13%; > 1 h: 16%; > 6 h: 21%; > 24 h: 27%). Oral anticoagulants prescription after the detection of atrial high-rate episodes lasting > 24 h was associated with a significant reduced stroke risk.

The retrospective study of Sandgren et al. [58] included 678 patients with pacemaker, with and without known AF. After a median follow-up of 38 months, the detection rate of subclinical AF was 30% in patients without known AF and 62% of these patients were prescribed oral anticoagulants. 80% of patients with known AF at pacemaker implantation already received oral anticoagulant therapy. The risk of stroke was 2.1% in patients with known AF at implantation, 1.9% in patients with detected subclinical AF and 1.4% in patients without AF or subclinical AF. The authors suggested that the risk of stroke in patients with detected subclinical AF may have been decreased by the relatively high prescription of oral anticoagulants after the initial detection of atrial high-rate episodes.

The above studies were able to demonstrate an association between oral anticoagulant therapy and a reduced risk of stroke.

In contrast to this, Marinheiro et al. [59] examined a study cohort of patients with pacemakers to assess the association between atrial high-rate episode detection (> 6 min) and a composite outcome including thromboembolic events and major bleedings. The primary outcome appeared in 4.9 per 100 person-year in the oral anticoagulants group compared to 3.4 per 100-person-year in the non-oral anticoagulants group. The investigators concluded that oral anticoagulant therapy does not lead to a significant difference in the risk of thromboembolic events and major bleedings in patients detected with atrial high-rate episodes.

The results of the LOOP study [56] have been long awaited. Svendsen et al. conducted a randomized controlled trial with 6004 patients, aged 70–90 years, without AF in history, and with at least one additional risk factor for stroke. Patients were randomized to insertable cardiac monitor group or control group including conventional follow-up for the detection of AF lasting > 6 min. After a mean follow-up of 64.5 months, the AF detection rates were 31.8% and 12.2% in the insertable cardiac monitor group and control group. Prescription of oral anticoagulants was initiated in 29.7% in the insertable cardiac monitor group versus 13.1% in the control group. Thromboembolic events and major bleedings appeared in 4.5% and 4.3% in the insertable cardiac monitor group compared to 5.6% and 3.5% in the control group. No statistically significant difference was found in either event, although the rate of oral anticoagulants prescription was significantly higher in the insertable cardiac monitor group.

The studies that investigated the prescription of oral anticoagulants in patients detected with subclinical AF/atrial high-rate episodes do not exhibit a clear benefit of the initiation of oral anticoagulant therapy (Table 4). The study of Perino et al. [57] indicated that the impact of oral anticoagulant therapy might depend on the subclinical AF/atrial high-rate episodes burden. One key limitation of the other studies is the lack of differentiation in the duration of subclinical AF/atrial high-rate episodes. Furthermore, the evidence is still low. Large prospective studies or randomized controlled trials as the recently published LOOP study [56] are required.

### Subclinical atrial fibrillation and atrial high-rate episodes, and the progression to clinical atrial fibrillation

There were several studies in the past that recorded an effect of the detection of subclinical AF/atrial high-rate episodes on the progression to a higher burden (Table 5).

**Table 5:** Subclinical atrial fibrillation and atrial high-rate episodes, and the progression to clinical atrial fibrillation

Study/first author (year)	Study design	*n*	Results
ASSERT (2012) [27]	Multicentre, prospective, randomized study	2580	Subclinical AF associated with 5.5-fold risk of clinical AF
Glotzer et al (2003) [29]	Analysis of the multicentre, prospective, randomized MOST trial	312	Patients with atrial high-rate episodes were 6 times as likely to develop clinical AF
Boriani et al (2018) [60]	Pooled analysis of 3 prospective observational studies	6580	49.8% of patients with atrial high-rate episodes transitioned to a higher AF burden; higher duration of atrial high-rate episodes and a CHA2DS2-VASc score ≥ 2 associated with a faster transition
Chen et al (2021) [38]	Retrospective cohort study	470	Highest association between atrial high-rate episodes lasting > 6 min to 24 h and clinical AF
Marinheiro et al (2019) [59]	Prospective observational study	923	Highest risk for clinical AF when atrial high-rate episodes lasted > 6 h
Park et al (2021) [44]	Prospective observational study	496	Progression to clinical AF was more likely when subclinical AF lasted at least 24 h
Witt et al (2015) [45]	Retrospective cohort study	394	Highest association between atrial high-rate episodes lasting > 24 h and the progression to clinical AF

*AF* atrial fibrillation, *CHA2DS2-VASc* congestive heart failure, hypertension, age ≥ 75 years, diabetes mellitus, stroke, vascular disease, age 65–74 years, sex category (female)

The ASSERT trial [27] demonstrated that subclinical AF was associated with a 5.5-fold risk of clinical AF. Glotzer et al. [29] were also able to demonstrate a similar increase in risk: patients with atrial high-rate episodes were 6 times as likely to develop AF.

Boriani et al. [60] analyzed data from three prospective studies of 6580 patients with cardiac implantable electronic devices and no history of AF to highlight AF burden and transition rates to higher burden. Among the patients with atrial high-rate episodes, 49.8% of patients transitioned to a higher AF burden. The higher the burden at diagnosis, the greater the likelihood of transition to a higher burden in the following 6 months (Fig. 3). Moreover, a higher duration of atrial high-rate episodes and a CHA2DS2-VASc score ≥ 2 were associated with a faster transition to a higher AF burden.

A recent study of Chen et al. [38] was also able to exhibit an association of the detection of atrial high-rate episodes and the progression to clinical AF. The authors specified the findings by demonstrating the highest association between atrial high-rate episodes lasting > 6 min to 24 h and subsequent AF. In addition, Marinheiro et al. [59] managed to narrow down the period more precisely by identifying the highest risk for future AF when atrial high-rate episodes lasted > 6 h. However, Park et al. [44] pointed out that a progression to clinical AF was more likely when subclinical AF lasted at least 24 h. Witt et al. [45] made the same observation: they demonstrated an association of atrial high-rate episodes and the progression to clinical AF and underlined that the association was strongest when atrial high-rate episodes lasted > 24 h.

Taken together, the studies that examined the association of atrial high-rate episodes detection and the progression to clinical AF were all able to demonstrate that the risk of a higher AF burden increased significantly with a higher duration of atrial high-rate episodes (Table 5). There is a clear positive correlation, but there is no clear cutoff value for the development of clinical AF. In view of the dynamic changes in the burden of subclinical AF/atrial high-rate episodes, it seems almost impossible to set a threshold value. Nevertheless, the findings might help to identify patients who are on a higher risk to develop AF and could, therefore, benefit from a closer follow-up with particular attention to a potential rising AF burden.

### The meaning of the CHA2DS2-VASc score in subclinical atrial fibrillation and atrial high-rate episodes

As described before, the current ESC guidelines recommend in patients with subclinical AF/atrial high-rate episodes detected by cardiac implantable electronic devices or insertable cardiac monitors a complete cardiovascular evaluation with ECG recording, clinical risk factors/comorbidity evaluation, and thromboembolic risk assessment using the CHA2DS2-VASc score [1]. In the past, there were studies that aimed to prove an association of the detection of subclinical AF/atrial high-rate episodes and the presence of a higher CHA2DS2-VASc score (Table 6).

**Table 6:** The meaning of the CHA2DS2-VASc score in subclinical atrial fibrillation and atrial high-rate episodes

Study/first author (year)	Study design	*n*	Results
Miyazawa et al (2021) [61]	Analysis of the multicentre, prospective, randomized IMPACT trial	2718	Atrial high-rate episodes lasting > 6 h more often in patients with high CHA2DS2-VASc score (≥ 3)
REVEAL AF (2017) [19]	Multicentre, prospective study	385	No association between subclinical AF and the CHA2DS2-VASc score
PREDATE AF (2017) [55]	Single-center, prospective study	245	No association between subclinical AF and the CHA2DS2-VASc score
Boriani et al (2018) [60]	Pooled analysis of 3 prospective observational studies	6580	Higher duration of atrial high-rate episodes and CHA2DS2-VASc score ≥ 2 associated with faster transition to higher AF burden
Miyazawa et al (2019) [31]	Retrospective cohort study	856	Adding atrial high-rate episodes to the CHA2DS2-VASc score improved the discrimination for thromboembolic events and death
Chen et al (2021) [36]	Retrospective cohort study	355	Risk for cardiovascular and cerebrovascular events increased with atrial high-rate episodes lasting > 30 s and higher CHA2DS2-VASc score (male: ≥ 2; female: ≥ 3)
Kaplan et al (2019) [42]	Retrospective cohort study	21,768	Increasing subclinical AF duration and CHA2DS2-VASc score associated with subsequent stroke; low stroke rates in patients with CHA2DS2-VASc score of 0 or 1 regardless the duration of subclinical AF
Kawakami et al. (2017) [62]	Retrospective cohort study	343	No association between atrial high-rate episodes and stroke when CHA2DS2-VASc score was low (0–2); association between atrial high-rate episodes and stroke when CHA2DS2-VASc score was ≥ 3

*AF* atrial fibrillation, *CHA2DS2-VASc* congestive heart failure, hypertension, age ≥ 75 years, diabetes mellitus, stroke, vascular disease, age 65–74 years, sex category (female)

Whereas Miyazawa et al. [61] showed that atrial high-rate episodes lasting > 6 h were more often in patients with a high CHA2DS2-VASc score (≥ 3), the REVEAL AF study [19] and the PREDATE AF trial [55] were not able to demonstrate an association. In these studies, the detection rates of subclinical AF were independent from the CHA2DS2-VASc score.

Boriani et al. [60] pointed out that the presence of a higher duration of atrial high-rate episodes and a CHA2DS2-VASc score ≥ 2 were associated with a faster transition to a higher AF burden. A retrospective study of Miyazawa et al. [31] investigated 856 patients with cardiac implantable electronic devices to assess the impact of atrial high-rate episodes on clinical outcomes such as thromboembolic events or death. The analysis revealed that atrial high-rate episodes were significantly associated with thromboembolic events or death and that adding atrial high-rate episodes as a risk factor to clinical risk scores such as the CHA2DS2-VASc score improved the discrimination for thromboembolic events and death.

Chen et al. [36] were also able to demonstrate that the combination of atrial high-rate episodes and the CHA2DS2-VASc score is useful in predicting cardiovascular and cerebrovascular events. The risk for cardiovascular and cerebrovascular events increased significantly with atrial high-rate episodes lasting > 30 s and a higher CHA2DS2-VASc score (male: ≥ 2; female: ≥ 3).

Kaplan et al. [42] used a database including 21,768 nonanticoagulated patients with cardiac implantable electronic devices to analyze the stroke risk after the detection of subclinical AF. They divided the patients into groups with no detected subclinical AF, subclinical AF lasting > 6 min to 23.5 h and subclinical AF lasting > 23.5 h. The authors illustrated that both an increasing subclinical AF duration and CHA2DS2-VASc score were associated with subsequent stroke. However, there were low stroke rates in patients with a CHA2DS2-VASc score of 0 or 1 regardless the duration of subclinical AF (Fig. 3). The study of Kawakami et al. [62] demonstrated similar results. They included 343 patients with pacemaker to evaluate the risk of thromboembolism when atrial high-rate episodes lasting > 6 min were detected. During a mean follow-up of 52 months, 48% of patients developed atrial high-rate episodes and 6% of patients suffered from stroke. Atrial high-rate episodes were significantly associated with stroke. A more in-depth analysis showed that there was no association between atrial high-rate episodes and stroke when the CHA2DS2-VASc score was low (0–2). However, atrial high-rate episodes and stroke were significantly associated when the CHA2DS2-VASc score was ≥ 3.

The studies were able to demonstrate an improvement of the value of subclinical AF/atrial high-rate episodes with the inclusion of the CHA2DS2-VASc score (Table 6). A trend is discernible that patients with a higher CHA2DS2-VASc score and, therefore, higher risk of thromboembolic events might benefit of a risk stratification strategy by combining the detection of atrial high-rate episodes and the CHA2DS2-VASc score. Additional insights could be gained from the analysis of the burden of subclinical AF/atrial high-rate episodes and their dynamics.

### Future directions and studies

An association between the detection of subclinical AF/atrial high-rate episodes and the risk of thromboembolic events has already been proven. Nevertheless, there is no clear causality. There are following questions that remain ambiguous: (1) which cutoff value of subclinical AF/atrial high-rate episodes detected by cardiac implantable electronic devices or insertable cardiac monitors is clinically significant? (2) Is there a difference in clinically significance in patients with cryptogenic stroke? (3) Does the prescription of oral anticoagulants reduce the risk of thromboembolic events and if so, from which duration of subclinical AF/atrial high-rate episodes would the patients benefit?

Another interesting question is whether screening for AF of the general population is appropriate and what the consequences would be. In the past few years, consumer wrist-worn wearable technologies such as smartwatches raised awareness. Using ECG technology or photoplethysmography, they are capable to detect an irregular heart rhythm [63–68]. The population-based Huawei Heart Study [68] examined 187,912 participants that used smart devices with photoplethysmography to monitor their pulse rhythm. AF was suspected in 424 participants, of whom 262 were actually followed up. 227 of 262 participants (87%) with suspected AF were confirmed as having AF (by health providers among the MAFA (mobile AF app) Telecare center and network hospitals, with clinical evaluation, electrocardiogram, or 24-h Holter monitoring). The positive predictive value of photoplethysmography signals was 91.6%, respectively. However, it should be noted that 38% of participants with suspected AF could not be effectively followed up, which would reduce the proportion of identified AF. The Apple Heart Study [66], including even 419,297 participants, aimed to prove an irregular pulse notification algorithm to detect possible AF in the general population. After a median monitoring time of 117 days, an irregular pulse was found in 2162 participants (0.52%). To confirm the diagnosis of AF, the participants concerned received ECG patches which should be worn for up to 7 days. Among the 450 participants with analyzable data from the ECG patches, 34% had AF. The use of smartwatches as a widely used screening tool for AF detection is controversial as the clinical significance is unclear. Furthermore, although the Huawei Heart Study [68] and the Apple Heart Study [66] were large population-based studies, their study cohorts were significantly younger (mean age was 34.7 years in the Huawei Heart Study and 41 years in the Apple Heart Study), reflecting smartwatch ownership. Nevertheless, wearables may identify individuals at high risk for AF due to early detection of irregular heart rhythm.

The value of systematic screening for AF in the general population is ambiguous. The STROKESTOP study [69] enrolled 28,768 individuals, aged 75 or 76 years, to either invited to screen for AF (*n* = 14,387) by 2 weeks of intermittent single-lead ECG monitoring or to the control group (*n* = 14,381). The slight risk increase in AF detection at 6 months was not persistent throughout the study period and, therefore, not statistically significant after a median follow-up time of 6.9 years. While a significant difference was demonstrated for the combined end point consisting of ischemic or hemorrhagic stroke, systemic embolism, bleeding leading to hospitalization, and all-cause mortality, this was not the case for ischemic stroke solely. Recently, the US Preventive Services Task Force states that evidence is lacking to assess the relation from benefits and harms of AF screening and anticoagulant therapy in case of screen-detected AF in the general population [70]. However, in the case of detection and confirmation of AF, an optimal risk evaluation and management of patients is important. As a basis for this, a structured characterization of AF patients (e.g., the 4S-AF scheme including stroke risk, symptom severity, severity of AF burden and substrate for AF) can be very helpful [71]. The simple Atrial fibrillation Care (ABC) pathway (’A’ for Anticoagulation/Avoid stroke; ‘B’ for Better symptom management; ‘C’ for Cardiovascular and Comorbidity optimization) provides integrated and comprehensive care of AF patients at all health care levels, to align the management and treatment approaches of generalists and specialists [1, 72].

There are three major pending studies that are expected to provide further insight regarding the clinical significance of subclinical AF/atrial high-rate episodes (Table 7). The ARTESIA study [73] examined if a treatment with apixaban, compared with aspirin, will reduce the risk of thromboembolic events in patients with device-detected subclinical AF and additional risk factors. The NOAH study [74] is an investigator-initiated, prospective, parallel-group, double-blind, randomized, multi-center trial. The aim is to demonstrate that oral anticoagulation using edoxaban is superior to current therapy to reduce the risk of thromboembolic events or cardiovascular death in patients detected with atrial high-rate episodes and at least two risk factors of stroke but without AF. The SILENT study [75] is a prospective, randomized study in patients with cardiac implantable electronic devices with sinus rhythm and a CHA2DS2-VASc score ≥ 2. The object of the study is to evaluate the impact of oral anticoagulant therapy on subclinical AF on the incidence of thromboembolic events.

**Table 7:** Future studies

Study	Study design	Estimated study completion date	Study cohort	Treatment/intervention arms	Primary endpoint
ARTESIA [63]	RCT	December 2023	SCAF detected by CIED or ICM	1. Apixaban 2. Aspirin	Composite of ischemic stroke and systemic embolism; major bleed
NOAH [64]	RCT	March 2022	AHRE detected by CIED or ICM	1. Edoxaban 2. Aspirin or placebo	Composite of ischemic stroke and systemic embolism; major bleed
SILENT [65]	RCT	October 2025	SCAF detected by CIED	1. Oral anticoagulant 2. No intervention	Stroke and systemic embolism

*RCT* randomized controlled trial, *SCAF* subclinical atrial fibrillation, *AHRE* atrial high-rate episodes, *CIED* cardiac implantable electronic device, *ICM* insertable cardiac monitor

## Conclusion

Continuous and long-term ECG monitoring using cardiac implantable electronic devices and insertable cardiac monitors revealed a high prevalence of subclinical AF/atrial high-rate episodes. The risk of thromboembolic events might increase with a higher subclinical AF/atrial high-rate episodes burden, particularly when the CHA2DS2-VASc score is high (Fig. 3). Although previous studies have not clearly demonstrated a benefit of oral anticoagulation in subclinical AF and atrial high-rate episodes, the ESC guidelines recommend risk stratification based on the duration of arrhythmias and the CHA2DS2-VASc score [1]. Nevertheless, the existing studies are not sufficient to understand the clinical impact of subclinical AF/atrial high-rate episodes. There is still a lack of evidence. Future studies could help to better assess the significance of detected subclinical AF/atrial high-rate episodes and the resulting consequences for clinical practice.
